# Subtractive Color Filters Based on a Silicon-Aluminum Hybrid-Nanodisk Metasurface Enabling Enhanced Color Purity

**DOI:** 10.1038/srep29756

**Published:** 2016-07-13

**Authors:** Wenjing Yue, Song Gao, Sang-Shin Lee, Eun-Soo Kim, Duk-Yong Choi

**Affiliations:** 1 Department of Electronic Engineering, Kwangwoon University, 20 Kwangwoon-ro, Nowon-gu, Seoul 01897, South Korea; 2 Laser Physics Centre, Research School of Physics and Engineering, Australian National University, Canberra ACT 0200, Australia

## Abstract

Highly efficient subtractive tri-color filters of cyan, magenta, and yellow with enhanced color purity and robustness have been proposed and realized, by exploiting a silicon-aluminum (Si-Al) hybrid-nanodisk (ND) metasurface atop a Si substrate. The aspect ratio of the Si-Al hybrid ND is much lower than that of the conventional Si nanowire, which is disadvantageous due to its fragility and low color purity. In response to incident light impinging upon the metasurface, the hybrid-NDs individually play the role in exciting a magnetic dipole (MD) resonance through the mediation of Mie-scattering between the hybrid ND and air. The light stored in the resonance is coupled to the substrate, giving rise to a suppressed reflection. By virtue of the top Al ND, the excited MD resonance is strongly confined by the Si ND. As a consequence, a near-zero resonant dip that exhibits high off-resonance reflection and narrow bandwidth is produced for embodying highly efficient tri-color filters with enhanced color purity. The spectral position can be tuned by a simple adjustment of the hybrid-ND diameter. A full-color palette was successfully created with a high color purity and large color gamut. The proposed devices may be applied for photorealistic high-resolution color printing and holographic displays.

Nanostructural color filters have attracted enormous attention as a viable alternative to the conventional dye-based colorants in a variety of applications including complementary metal-oxide-semiconductor (CMOS) image sensors, photorealistic high-resolution color printing, three-dimensional and multicolor holographic displays, and photovoltaic devices^1^,^2^,^3^,^4^,^5^,^6^,^7^. By capitalizing on a Fabry-Perot etalon or a subwavelength grating structure that invokes either the plasmonic resonance or the guided-mode resonance, noble metals like gold and silver are mostly relied on for the previous schemes^8^,^9^,^10^,^11^,^12^,^13^,^14^, which are critically dependent on their fidelity with fine nanoscale structures and the possibility of integration with other photonic/electronic devices. Silicon (Si), known as the second-most-abundant material in nature, has been predominantly adopted in the commercial microelectronics industry. Owing to its salient features, such as a low cost, compatibility with the existing CMOS processing, and a low loss in the visible spectral band, Si is regarded as the best candidate for the construction of nanostructural color filters. It was reported that a Si nanowire (NW) formed on a Si substrate is suitable for multi-color generation, whereby a wavelength-selective coupling of the incident light with the guided modes of a vertical Si NW renders a specific color^15^,^16^,^17^,^18^. For the Si NW, however, the height and diameter are typically in the range of several micrometers and tens of nanometers, respectively, resulting in the exhibition of an extremely large aspect ratio; therefore, the Si NW device is inevitably susceptible to untenable fragility and an unstable performance that is incurred by the bunching of the neighboring NWs^19^. In addition, the reflection spectra of the device tend to induce a single non-zero reflection dip at resonance^15^ or multiple resonant dips^17^,^18^ in the visible band, severely degrading the color purity.

From the perspective of the embodiment of a robust, ultrathin color filter, a lattice of Si nanodisks (NDs) featuring a low aspect ratio is categorically preferred to an array of elongated NWs. In contrast with the conventional materials with predetermined intrinsic properties, a Si-ND array, functioning as a dielectric metasurface, could be flexibly designed to deliver customized optical properties such as a negative refractive index and optical cloaking^20^,^21^,^22^,^23^,^24^,^25^. Such a dielectric metasurface is rarely vulnerable to conduction loss, unlike plasmonic metallic nanostructures^25^,^26^,^27^,^28^,^29^,^30^,^31^,^32^,^33^. Each of the Si ND elements is deemed to support a Mie-scattering-mediated resonance in the form of the electrical dipole (ED) and the magnetic dipole (MD), which is effectively tuned by adjusting the ND diameter. A broadband antireflection coating capitalizing on a Si metasurface was suggested for the trapping of light in a solar cell, where the incident light is resonantly channeled to the Si substrate so that a reflection is satisfactorily prevented^26^,^33^. It should be remarked that in view of the high color purity a reflective subtractive color filter is required to provide a narrow, near-zero resonant dip in conjunction with a high off-resonance reflection efficiency.

In this paper, we propose and demonstrate highly efficient structural subtractive tri-color filters of cyan, magenta, and yellow (CMY) that feature an enhanced color purity and robustness, taking advantage of a Si-Al hybrid-ND metasurface that is formed on a Si substrate. Each of the hybrid-ND elements that constitute the metasurface is assigned the central role of supporting an MD-based resonance mode that is induced by Mie-scattering. The excited MD mode is efficiently confined by the Si ND, which is facilitated by the Al ND at the top. A near-zero resonant reflection dip, which provides a high off-resonance reflection efficiency and a pertinent bandwidth, is obtained to embody the tri-color filters featuring the enhanced color purity. For the reflection dip, the underlying mechanism was understood by inspecting the electric- and magnetic-field profiles pertaining to the Si-Al hybrid-ND elements, while the position was tuned throughout the visible band by particularly altering the ND diameter. To verify the claimed performance in terms of the full-color generation, enhanced color purity, extended color gamut, and a high reflection, a full-color palette was successfully prepared by incorporating an array of filter elements with a span of ND diameters and periods.

## Results

### Subtractive tri-Color Filters with an Enhanced Color Purity

**Figure 1:**
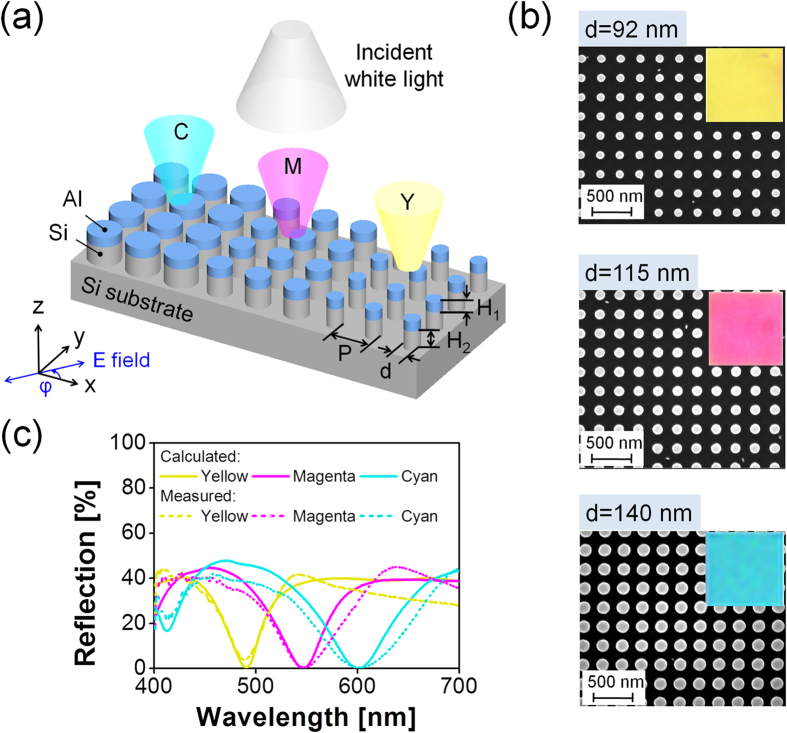
Proposed subtractive CMY color filters incorporating a Si-Al hybrid-ND metasurface formed on a Si substrate. (**a**) Schematic configuration. (**b**) SEM images of the fabricated color filters with diameters of d = 92 nm, 115 nm, and 140 nm for a fixed period of P = 240 nm, corresponding to yellow, magenta, and cyan, respectively. Inset reveals individual optical microscope images produced by the filters. (**c**) Reflection spectral responses of the CMY devices for normal incidence.

Figure 1(a) shows the schematic configuration of the proposed subtractive CMY color filters sitting on a Si substrate, taking advantage of a square lattice comprising the integration of Si NDs with Al NDs. For the Si-Al hybrid-ND array, referring to the combination of the bottom Si NDs and the top Al NDs, the period (P) is identical along the x and y directions while the heights of the Al and Si layers are H_1_ and H_2_, respectively. The polarization of the normally incident light is indicated by an angle φ of the electric (E) field with respect to the x direction. A metasurface consisting of the Si-Al hybrid NDs is supposed to exhibit a reflection dip that is a result of a strong wavelength selective resonance. The reflection dip can be tuned by appropriately tailoring the ND diameter, thereby producing the three representative subtractive CMY colors. For the manufactured CMY filters, the period is P = 240 nm, while the heights of the Al and Si NDs are H_1_ = 50 nm and H_2_ = 180 nm, respectively. It is noted that less fabrication steps are required here as the Al-ND pattern is used as a hard etch mask for the Si NDs, which does not need to be removed at the last stage, as was required for the previous approaches^15^,^16^,^17^,^18^. Figure 1(b) shows the scanning electron microscope (SEM) images of the fabricated devices, wherein three different types of well-defined circular ND patterns that correspond to the diameters of d = 92 nm, 115 nm, and 140 nm are presented from top to bottom. The inset of the SEM images shows the bright-field optical microscope image of the color filters with dimensions of 30 × 30 μm^2^ that comprises vivid and bright colors of yellow, magenta, and cyan. The characteristics of the calculated and measured reflection spectra are plotted in Fig. 1(c), where a high correlation between the calculated and measured results was obtained. Observations indicate that the proposed color filters exhibit a near-zero resonant reflection dip, while efficiently reflecting back incident light in off-resonance regions, which is desirable for the improvement of the color purity. Because of the presence of the optically thick Si substrate, it is presumed that the proposed filter does not allow for any transmission, and it potentially acts as a near-perfect absorber.

### Color tuning and demonstration of a vivid full-color palette

**Figure 2:**
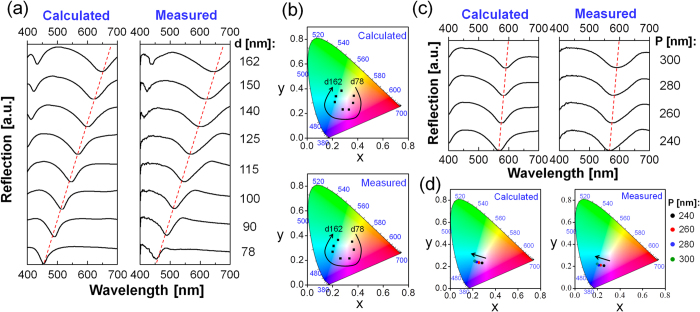
ND diameter- and period-dependent reflection spectra and corresponding color responses. (**a**) Reflection spectra of the proposed filters with diameters ranging from d = 78 to 162 nm for a fixed period of P = 240 nm, with the red dashed line tracing the location of the reflection dip. (**b**) Chromaticity coordinates in the CIE 1931 chromaticity diagram corresponding to the spectra depending on the ND diameter. (**c**) Reflection spectra in response to the period ranging from 240 nm to 300 nm for a fixed diameter of d = 120 nm, with the locus of the reflection dips traced in red dashed line. (**d**) Chromaticity coordinates corresponding to the spectra as a function of the period.

The proposed subtractive color filter is capable of scanning the resonance wavelength throughout the visible band by tailoring the diameter of the Si-Al hybrid ND. Figure 2(a) shows the calculated and measured reflection spectra as the ND diameter increases from d = 78 nm to 162 nm for a constant period of P = 240 nm. The reflection dip, traced by a red dashed line, appears to red shift from λ = 453 nm to 648 nm with an increasing diameter. A plotting of the corresponding chromaticity coordinates in a standard International Commission on Illumination (CIE) 1931 chromaticity diagram is featured in Fig. 2(b). According to the trace of the plotted chromaticity coordinates, a palette of vivid full colors is attained through the adjustment of the ND diameter. As shown in Supplementary Figure S1, a linear relationship is confirmed between the resonance wavelength and the ND diameter for a constant gap of 100 nm. The reflection characteristics were then explored through the changing of the ND period from P = 240 to 300 nm for a constant diameter of d = 120 nm under the normal incidence. As shown in Fig. 2(c), the reflection dip that is traced by the red dashed line slightly red shifted with an increasing period; meanwhile, it is noted for the case of conventional Si nanowires that the reflection dip is almost independent of the period, which ranges up to several micrometers^15^. Considering that the periods of the proposed Si-Al hybrid-ND array in the subwavelength regime are much smaller, the near-field coupling between the adjacent NDs might account for the slight red shift in the resonance wavelength with the increasing period. A new resonance dip will appear at the wavelength that is determined by the period in accordance with the Rayleigh anomaly, which is related to a periodic grating structure, the period of which is comparable to the wavelength of concern^26^,^32^. To permit only a single primary resonance dip within the visible band, an essentially subwavelength period should be applied. Figure 2(d) depicts the chromaticity coordinates on the basis of the calculated and measured reflection spectra with the period, implying that the color is nearly stable with respect to the period, as opposed to a case where the ND diameter is adjusted; furthermore, it is confirmed from Supplementary Figure S2 that the proposed subtractive color filters are capable of providing polarization-independent properties and stable optical performances for incident angles ranging up to 26°.

**Figure 3:**
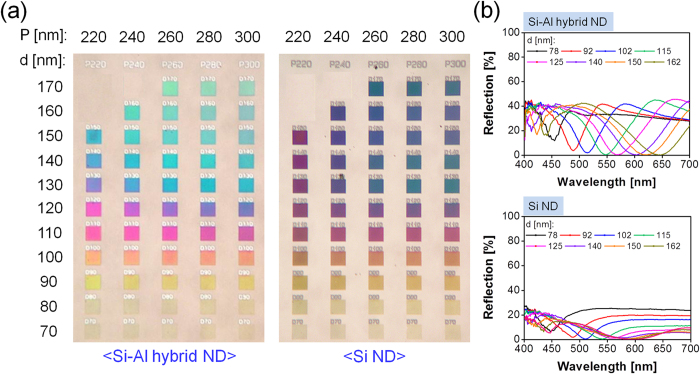
Bright-field microscope images and measured reflection spectra of manufactured color palettes. (**a**) Measured bright-field microscope images of the prepared color palette based on the proposed Si-Al hybrid-ND metasurface and the Si ND case. Each filter consists of NDs with different diameters and periods, having a footprint of 30 × 30 μm^2^, and (**b**) measured reflection spectra for the proposed Si-Al hybrid-ND-based filters and the Si ND case, as the diameters increase from 78 nm to 162 nm for a period of P = 240 nm.

We strived to construct a vivid full-color palette by taking advantage of an array of filter elements with a span of ND diameters and periods. The corresponding bright-field optical microscope images are displayed in Fig. 3(a), where each filter has a footprint of 30 × 30 μm^2^. Given the color images that were taken after the removal of the top Al ND, the color palette that is based on the Si-Al hybrid ND was checked to yield an enhanced performance in terms of the color purity and brightness, visual contrast, and color gamut. A continuum of color ranging from yellow to green was attained by increasing the ND diameter from d = 70 nm to 170 nm for a constant period, as was intended. For a fixed ND diameter, it was revealed that the resulting color is not profoundly susceptible to variations of the period. To investigate the dependence of the produced color on the top Al ND that belongs to the Si-Al hybrid ND, as shown in Fig. 3(b), the reflection spectra were recorded with respect to the diameter for a constant period of P = 240 nm. In the presence of the Al ND, the spectral shape sharpened decently, and the off-resonance reflection exhibited a nearly two-fold efficiency increase from 20 to 40%, proving that the color purity and brightness can be enhanced for the proposed color filter. For an array of Si NDs formed on a Si substrate, the off-resonance reflection may be predominantly determined by the relatively low reflectivity of Si. Considering a metallic Al film with a 50-nm thickness exhibits a reflectivity that is over two times as high as that of Si, it is thought that for the proposed filter based on the Si-Al hybrid ND, the nearly two-fold increase in the off-resonance reflection is mainly attributed to the substantially enhanced reflectivity which results from the presence of Al instead of Si. When the diameter of the Si-Al hybrid ND varied from d = 78 nm to 162 nm, the reflection dip was readily tuned over a broad spectral range from λ = 453 nm to 648 nm, translating into a wide color tunability

### Mechanism for the wavelength selective reflection dips

**Figure 4:**
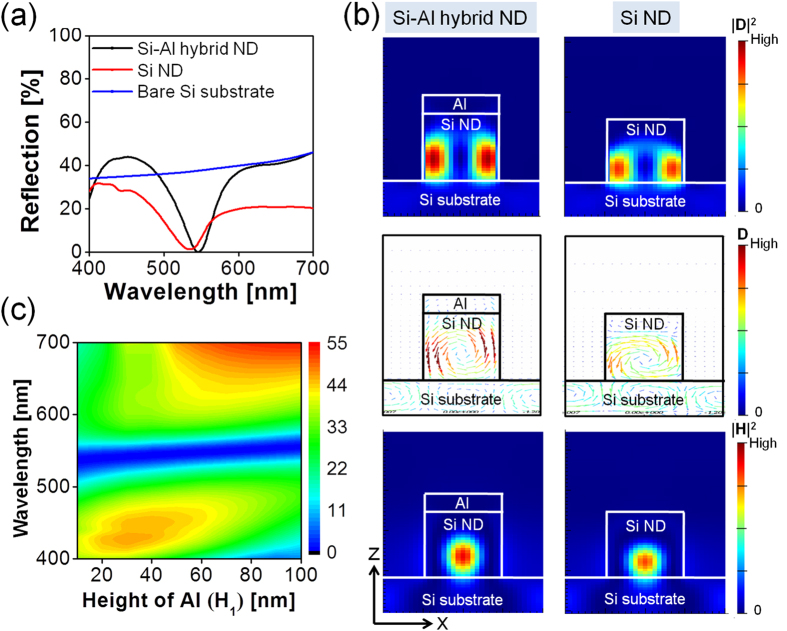
Comparison of reflection spectra, field profiles at resonance and dependence of the reflection spectra of proposed color filters on the height of top Al ND. (**a**) Calculated reflection spectra for the proposed magenta filter, Si-ND structure, and bare Si substrate. (**b**) D-field intensity profile (|D|^2^) and the corresponding vector plot, and the H-field intensity profile (|H|^2^) for the proposed filters at resonance, in comparison to the case of the Si-ND array; here, a set of circular D-field loops develop, whereby they underlie the MD resonance modes for both cases. (**c**) Contour map of the reflection spectra of the proposed filters, with the height of the Al ND ranging from H_1_ = 10 nm to 100 nm.

The task of exploring the underlying mechanism of the resonant reflection dip for the proposed color filters for which a Si-Al hybrid-ND metasurface is drawn upon was also undertaken. Each of the Si NDs, serving as a nanoscatterer of a high refractive index, is capable of exciting both MD and ED resonances in the visible band. The MD resonance is relevant to an electric displacement current loop that develops inside the Si ND and occurs at the wavelength in the proximity of the Si ND diameter. The electromagnetic properties of the dielectric metasurface are conspicuously governed by the structural parameters of the scattering elements such as the Si ND^30^, reflecting the fact that the resonance wavelength is principally dependent on the ND diameter. With the Si-ND-based dielectric metasurface tethered to the Si substrate, the light stored in the ND resonator can preferably couple to the high-index substrate, and this accounts for the drastically reduced reflection. The spectral bands for the ED and MD modes will eventually merge to form a single broad band^26^,^28^. Figure 4(a) shows the results of a comparison of a Si-ND diameter of d = 100 nm with a bare Si substrate. For the proposed filter, for which a Si-Al hybrid-ND metasurface with the same diameter is incorporated, the reflection is weakened at a resonance wavelength that is similar to the case of a Si ND metasurface, indicating that the supported resonance is basically identical for the two cases. In a bid to further validate the excitement of the resonance for the proposed case, as shown in Fig. 4(b), the intensity profiles of the electric flux displacement D-field and the magnetic H-field were monitored with the incident E-field aligned along the x-axis (φ = 0°), for a comparison with the case of a Si ND metasurface. We obtained similar field profiles for the two cases and concluded that the Al ND on top of the Si ND negligibly affects the original resonance mode that is supported by the Si ND-based dielectric metasurface. Through observations, it was evident that the D-field intensity profile (|D|^2^) and the corresponding vector plot form a circular loop, indicating an out-of-plane MD resonance, which is implied from the H-field intensity profile (|H|^2^). The ED resonance is invisible in the case of a high-index Si substrate, unlike the free-standing Si ND^28^,^33^, proving that the operation of the proposed filters is chiefly governed by the MD resonance. For the Si ND metasurface, the MD resonance mode appears to be weakly confined, exhibiting a relatively large overlap with the high-index Si substrate. A broad spectral bandwidth is accordingly obtained as implied in Fig. 4(a). For the Si-Al hybrid-ND metasurface associated with the proposed filter, however, it is observed the top Al ND plays a role of strengthening the confinement of the MD resonance mode within the Si ND, with the excited MD resonance mode displaced upward away from the substrate. Noting the stronger the mode confinement, the narrower the spectral bandwidth, it is confirmed that the proposed filter involving a top Al ND is capable of providing a remarkably narrow reflection band, as desired. Figure 4(c) depicts the influence of the height of the Al ND (H_1_) on the reflection spectra, whereby the reflection dip at resonance that is marked in blue remains stable regardless of the height H_1_, which is varied from 10 nm to 100 nm. In contrast to the case of the plasmonic metasurface, suffering from its high sensitivity to the thickness of the metallic grating^6^, the height-independent resonance suggests that the operation of the filters capitalizing on the Si-Al hybrid-ND metasurface is attributed to the Mie-scattering-mediated MD resonance that is accommodated by the Si ND.

## Discussion

In summary, highly efficient subtractive CMY tri-color filters that enable an enhanced color purity and robustness have been presented, for which a Si-Al hybrid-ND metasurface formed on a Si substrate. It was discovered that the strongly suppressed reflection that is responsible for a near-zero dip is primarily ascribed to the MD resonance that is supported by hybrid-ND elements. With the aid of the Al ND, the color purity has been profoundly enhanced as a result of the near-zero reflection dip at resonance, in conjunction with the high off-resonance reflection and a narrow bandwidth. The resonance dip corresponding to a specific color could be tuned throughout the visible band by virtue of adjusting the hybrid-ND diameter. Besides the manufactured CMY tri-color filters, a vivid full-color palette, consisting of an array of filter elements with different ND diameters and periods, was realized to provide a high performance in terms of the color purity and brightness, visual contrast, and color gamut. It is anticipated that the proposed approach will be readily made based on other high index dielectric materials so that it can be applied for photorealistic high-resolution color printing and holographic displays.

## Methods

### Numerical Simulation

Simulations of the reflection spectra were performed by using a simulation tool based on the finite difference time domain (FDTD) method (FDTD Solutions, Lumerical, Canada). The dispersion characteristics of the materials that encompass Al and Si were derived from the multi-coefficient model offered by the simulation tool. Simulations were performed with a plane wave under normal incidence.

### Device Fabrication

The proposed subtractive color filters were manufactured with a footprint of 30 × 30 μm^2^. A square lattice of holes were initially patterned on a single crystalline Si wafer using an electron-beam lithography (EBL) system (RAITH 150) with ZEP520A postitive electron-beam resist. A 60-nm thick Al layer was then deposited with an electron-beam evaporator (Temescal BJD-2000 E-beam Evaporator system). A group of Al NDs was formed after the lift-off process with ZEP remover (ZDMAC). The Si substrate masked by the Al ND pattern was meticulously dry etched in a plasma etcher (ICP-RIE Plamalab100 from Oxford) using a mix of CHF_3_ and SF_6_ gases to complete the Si ND of a desired height.

### Optical Characterization

A cross-sectional structure of the fabricated CMY color filter were observed under a high resolution scanning electron microscope (UltraPlus analytical FESEM, Zeiss), as shown in Fig. 1(b). The characteristics of the reflection spectra were practically inspected under the normal incidence using a spectroscopic reflectometer (Elli-Rsc, Ellipso Technology).

## Additional Information

**How to cite this article**: Yue, W. *et al*. Subtractive Color Filters Based on a Silicon-Aluminum Hybrid-Nanodisk Metasurface Enabling Enhanced Color Purity. *Sci. Rep.* **6**, 29756; doi: 10.1038/srep29756 (2016).

## Supplementary Material

Supplementary Information
