# Supplementary material for: Subtractive Color Filters Based on a Silicon-Aluminum Hybrid-Nanodisk Metasurface Enabling Enhanced Color Purity
Source: Sci Rep. 2016 Jul 13;6:29756. doi: 10.1038/srep29756 (PMC4942830; doi:10.1038/srep29756)
Supplement: Supplementary Information [file srep29756-s1.pdf]

Supplementary Information

Subtractive Color Filters Based on a Silicon-Aluminum Hybrid-Nanodisk Metasurface Enabling Enhanced Color Purity

Wenjing Yue[1], Song Gao[1], Sang-Shin Lee[1],* Eun-Soo Kim[1], and Duk-Yong Choi[2]

[1]Department of Electronic Engineering, Kwangwoon University, 20 Kwangwoon-ro, Nowon-gu, Seoul 01897, South Korea

[2]Laser Physics Centre, Research School of Physics and Engineering, Australian National University, Canberra ACT 0200, Australia

1. Dependence of the location of a reflection dip on the ND diameter

Supplementary Figures S1(a) and (b) depict the contour map of the calculated reflection spectra as a function of the ND diameter at a constant gap of 100 nm that is between the adjacent NDs, for the proposed filter based on the Si-Al hybrid-ND metasurface and the case of the Si NDs, respectively. For both cases, the location of the reflection dip, marked by a white dashed line, is observed to red shift linearly as the diameter increases from d=70 nm to 170 nm. Compared with the Si ND metasurface, the reflection spectra of the proposed filters are recognized by a narrow, near-zero reflection dip in conjunction with high reflection at off-resonance. The spectral tunability that is mediated by the ND diameter may translate into a full-color generation.

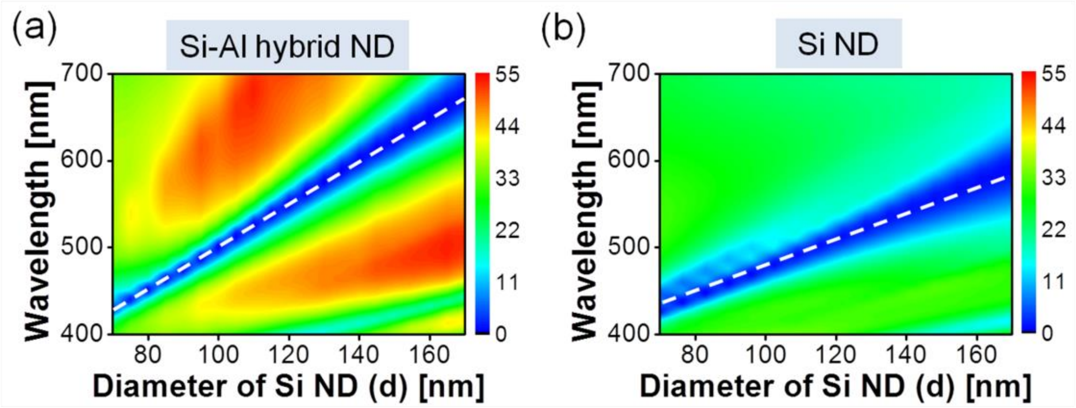

Figure S1. Contour map of the calculated reflection spectra for (a) the proposed Si-Al hybrid-ND-based filters, and (b) the case of the Si-ND-based

structure, with the diameter varying from 70 nm to 170 nm for a constant gap of 100 nm.

2. Polarization and angle dependent characteristics of the proposed color filters

For the representative CMY color filters, the calculated reflection spectra were carefully inspected for different polarization directions of φ=0°, 45°, and 90°, as has been plotted in Supplementary Figure S2(a). Equivalent reflection spectra could be obtained for the devices, with reflection dips located at λ=490 nm, 546 nm, and 600 nm, respectively. The contour map of the spectra that are associated with the proposed magenta filter is plotted in terms of the incident angle, as shown in Supplementary Figure S2(b). The calculated reflection spectra are observed to lead to an approximately invariant resonance position and the near-zero reflection, which is marked by the white dashed line, for an angle ranging up to 26°.

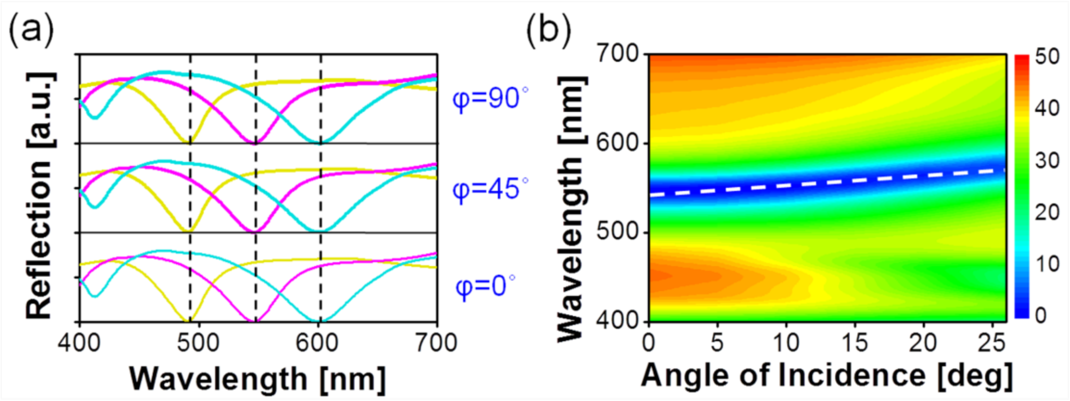

Figure S2. (a) Calculated reflection spectra of the proposed subtractive CMY color filters for different polarization directions including φ=0°, 45°, and 90°. (b) Contour map of the spectra of the magenta color filter with the incident angle, where the resonance dip is traced by a white dashed line.
